# Unrecognized myocardial infarctions assessed by cardiovascular magnetic resonance are associated with the severity of the stenosis in the supplying coronary artery

**DOI:** 10.1186/s12968-015-0202-5

**Published:** 2015-11-19

**Authors:** Per Hammar, Anna M. Nordenskjöld, Bertil Lindahl, Olov Duvernoy, Håkan Ahlström, Lars Johansson, Nermin Hadziosmanovic, Tomas Bjerner

**Affiliations:** Västmanland County Hospital Västerås, Department of Radiology, Västerås, S-72189 Sweden; Department of Cardiology, Örebro University Hospital, S-70182 Örebro, Sweden; Uppsala Clinical Research Centre, S-75237 Uppsala, Sweden; Department of Medical Sciences, Cardiology, Uppsala University, Uppsala, S-75105 Sweden; Department of Radiology, Oncology and Radiation Science, Uppsala University, S-75185 Uppsala, Sweden; Astra Zeneca, Molndal, Sweden

**Keywords:** Angiography, Coronary disease, Imaging, Infarction, Cardiovascular magnetic resonance

## Abstract

**Background:**

A previous study has shown an increased prevalence of late gadolinium enhancement cardiovascular magnetic resonance (LGE CMR) detected unrecognized myocardial infarction (UMI) with increasing extent and severity of coronary artery disease. However, the coronary artery disease was evaluated on a patient level assuming normal coronary anatomy. Therefore, the aims of the present study were to investigate the prevalence of UMI identified by LGE CMR imaging in patients with stable angina pectoris and no known previous myocardial infarction; and to investigate whether presence of UMI is associated with stenotic lesions in the coronary artery supplying the segment of the myocardium in which the UMI is located, using coronary angiography to determine the individual coronary anatomy in each patient.

**Methods:**

In this prospective multicenter study, we included patients with stable angina pectoris and without prior myocardial infarction, scheduled for coronary angiography. A LGE CMR examination was performed prior to the coronary angiography. The study cohort consisted of 235 patients (80 women, 155 men) with a mean age of 64.8 years.

**Results:**

UMIs were found in 25 % of patients. There was a strong association between stenotic lesions (≥70 % stenosis) in a coronary artery and the presence of an UMI in the myocardial segments supplied by the stenotic artery; it was significantly more likely to have an UMI downstream a stenosis ≥ 70 % as compared to < 70 % (OR 5.1, CI 3.1-8.3, *p* < 0.0001). 56 % of the UMIs were located in the inferior and infero-lateral myocardial segments, despite predominance for stenotic lesions in the left anterior descending artery.

**Conclusion:**

UMI is common in patients with stable angina and the results indicate that the majority of the UMIs are of ischemic origin due to severe coronary atherosclerosis. In contrast to what is seen in recognized myocardial infarctions, UMIs are predominately located in the inferior and infero-lateral myocardial segments.

**Trial registration:**

The PUMI study is registered at ClinicalTrials.gov (NCT01257282).

**Electronic supplementary material:**

The online version of this article (doi:10.1186/s12968-015-0202-5) contains supplementary material, which is available to authorized users.

## Background

Myocardial infarction (MI) is typically associated with symptoms (e.g. chest pain) that cause the patient to seek medical care. However, MI might be asymptomatic or have atypical symptoms that are not recognized by either the patient or the health professionals as indicating MI. These silent myocardial infarctions or more correctly, unrecognized myocardial infarctions (UMIs), have traditionally been detected as pathological Q-waves on electrocardiography (ECG) taken after the acute event. UMIs represent between 22 and 44 % of all myocardial infarctions [1]. In the general population the prevalence of UMI detected by Q-waves is strongly related to age. It is extremely rare under the age of 40, while the prevalence is > 5 % in men >75 years of age [2]. The mortality rates after unrecognized and recognized myocardial infarctions are similar [3]*.*

Late gadolinum enhancement cardiac magnetic resonance (LGE CMR) is a recent technique that can identify even very small MIs [4]. Using this technique previous studies have shown a prevalence of UMIs among 65–75 -year-olds in the general population of 17–30 % [5–7]. UMI was more than four times more common than recognized myocardial infarctions (RMIs), 19.8 % and 4.4 %, respectively, in the study of Barbier et al. [7].

Kim et al. have recently shown that the prevalence of LGE CMR detected UMI increased with the extent and severity of coronary disease in the patient [8]*.* However, to the best of our knowledge, the association between the severity of atherosclerosis in a specific coronary artery and occurrence of an UMI in the myocardial segments supplied by that very same artery has not previously been investigated. Therefore, the aims of the present study were twofold: to investigate the prevalence of UMI detected by LGE CMR imaging in patients with stable angina pectoris and no known previous MI; and to elucidate whether UMI is associated with stenotic lesions in the coronary artery supplying the segment of the myocardium in which the UMI is located.

## Methods

### Study population

In the prospective multicenter study, Prevalence and Prognostic Value of Unrecognized Myocardial Injury in Stable Coronary Artery Disease (PUMI), we included patients with angina pecoris scheduled for coronary angiography. The diagnosis of angina pectoris was made by the treating physician based on symptoms compatible with stable angina pectoris. The decision to perform coronary angiography was made prior to study recruitment. In all patients included in the study a LGE CMR examination was scheduled for research purpose only prior to the coronary angiography. In the normal clinical work-up of patients with symptoms of stable angina pectoris at our institutions, no LGE CMR examinations are usually done. Exclusion criteria were: pathological Q-wave on 12-lead ECG, previously known myocardial infarction, previous percutaneous coronary intervention (PCI) or coronary artery bypass graft (CABG), history of congestive heart failure, estimated glomerular filtration rate (GFR) < 30 ml/min/1,73 m^2^, conventional conditions contraindicating an CMR examination (e.g. pacemaker, claustrophobia, intracranial clips) or lack of suitability for participation in the study for any reason as judged by the investigator. Patients were enrolled from 6 Swedish sites: Danderyd County Hospital (*n* = 13), Falun County Hospital (*n* = 68), Gävle County Hospital (*n* = 22), Linköping University Hospital (*n* = 32), Uppsala University Hospital (*n* = 87) and Örebro University Hospital (*n* = 43) during the period January 2008 to March 2011.

**Fig. 1:**
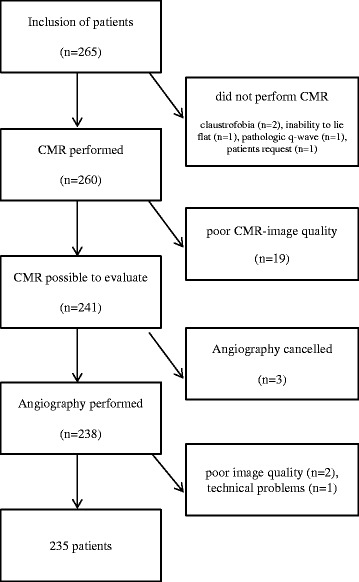
Graphical description of how the study cohort of 265 patients resulted in 235 evaluable subjects. CMR = cardiovascular magnetic resonance

Of the 265 included patients in the study, the 235 patients that had both a coronary angiography and a CMR possible to analyse constituted the study cohort in the present report (Fig. 1). Five patients did not perform CMR, 19 patients were excluded because of poor MR quality, six patients either did not perform coronary angiography or was not possible to evaluate.

The study was approved by the regional ethics committee in Uppsala (U-07-001 PUMI) and all patients provided written informed consent. The PUMI study is registered at ClinicalTrials.gov (NCT01257282).

### Study procedure

Patients referred for coronary angiography to the participating hospitals were screened for inclusion and exclusion criteria. In eligible patients enrolled, after obtaining the clinical history and a physical examination, LGE CMR was performed. In the study population we performed LGE CMR imaging prior to, but not more than 4 weeks before, the scheduled coronary angiography.

During the study the patients received treatment at the discretion of the responsible physician.

### Image acquisition

CMR was performed on clinical 1,5-T scanners (Philips Intera, Best, the Netherlands; Philips Achieva, Best, the Netherlands or Siemens Symphony, Erlangen, Germany) using a general scanning protocol. This consisted of survey scans to plan further scanning, cine short axis images and a viability sequence in short axis (SA), long axis 2-chamber, 3-chamber and 4-chamber views using ECG-triggering and breath-holding. Patients were given an injection of 0.15 ml/kg bodyweight (maximum dose 15 ml) of gadobutrol (Gadovist®, Bayer, Leverkusen, Germany) and viability imaging was performed with a minimum delay of 15 minutes. In the waiting time cine short axis was performed. This consisted of a steady state free precession (SSFP) sequence with the following parameters: repetition time (TR) shortest, echo time (TE) shortest, flip angle 70°, image matrix 176x190 reconstructed to a voxel size 1.37x1.37x6 mm with a slice gap of 4 mm, 18 heart phases acquired, 2 slices per breath hold. The viability sequence was a 3D inversion recovery gradient echo sequence with the following parameters: TR set to shortest (typically 4.0-4.2 ms), TE set to shortest (typically 1.18-1.28 ms), inversion time (TI) chosen by the operator to null normal myocardium, flip angle 15°, image matrix 256x100, FOV 375 x 281 mm, acquired voxel size 1.46x2.81x10 mm reconstructed to a voxel size 0.73x0.73x5 mm. Eleven slices were acquired per breath hold for the long axis slices (2CH, 3CH and 4CH) and 22 slices divided in two breath holds for the short axis. Each breath hold were 16 seconds at heart rate 60 bpm. A 3D sequence was used instead of 2D contiguous slices. This strategy allows imaging in 4 different planes with good patient compliance due to less breath-holds and shorter examination time. This protocol was acquired in the majority of the subjects. A few sites had to adapt the protocol (e.g. due to hardware constraints) to ensure best possible image quality to demarcate LGE. In the single site with Siemens equipment 2D imaging with contiguous slices was used due to difficulties with applying 3D imaging at that site. When this had to be done the adaption was chosen to be as minor as possible compared to the standard protocol.

Coronary angiography was performed after (more than 12 hours but less than 4 weeks) the CMR in a routine way with standard projections.

### Image analysis, CMR

At CMR, areas of LGE that were visible in at least two imaging planes were noted and localized using the American Heart Association (AHA) 17-segment model, proposed by Cerqueira et al. [9]. The images were analysed at the core lab by two radiologists (T.B. and P.H.) in consensus. Areas of LGE could engage one or several adjacent segments. If there were distinctly separate areas of LGE, each of those was assessed individually. Presence of LGE in each subject was noted. If a LGE had a subendocardial component it was categorized as “transmural” or “subendocardial” depending on whether the contrast enhancement was reaching the epicardium or not [10]. In the following, subjects within those two groups are labelled unrecognized myocardial injury (“UMI”). Areas of LGE without a subendocardial component, i.e. located only subepicardially or in the center of the myocardium, were labelled as “no MI” together with subjects with no LGE. The myocardium displaying LGE was analysed using manual contouring of the area in each short axis slice on a clinical PACS-system (Carestream Health, Rochester, NY, USA) and the volumes of the enhanced myocardium were calculated. Finally UMI mass was calculated by multiplying the volume by the density of the myocardial tissue (1.05 g mL^−1^).

The treating physician had no access to the results of the analysis of the CMR examination with the exception of calculated ejection fraction (EF) and any observed wall motion abnormalities.

### Image analysis, coronary angiography

CMR was followed by coronary angiography. In accordance with the Swedish Coronary Angiography and Angioplasty Registry (SCAAR) the coronary vessels were divided in 19 segments, derived from the 16 segment model proposed by Austen et al. [11].

The degree of narrowing of the diameter in each of the 19 coronary segments was visually categorized as 0-29 %, 30-49 %, 50-69 %, 70-99 % or 100 % (occlusion) in accordance with the SCAAR-registry. This methodology is in accordance with previous published studies on the SCAAR-registry [12].

In the proposed AHA 17-segment model by Cerqueira et al. [9] individual myocardial segments are assigned to specific coronary arteries. However, this assumption has been challenged and has been shown to be inaccurate [13, 14], e.g. Ortiz-Pérez showed in a CMR-study that only four segments were completely specific for the left anterior descending artery (LAD), but no segments were completely specific for the right coronary artery (RCA) or for the circumflex artery (LCX) [14]. Therefore, in each patients we assessed visually for all coronary arteries with a stenosis of ≥30 %, which of the myocardial segments in the 17-segment model that were supplied by that particular artery downstream the stenosis, taking the individual coronary anatomy in consideration. This assessment was done at the core lab by two radiologists (P.H. and O.D.) blinded for the result on CMR, first individually, then in consensus.

### Analysis of association between coronary artery stenosis and LGE

The association between coronary artery stenosis and presence of LGE was analysed in two ways. In the primary analysis, a perfect match was required between the myocardial segments deemed to be supplied by a coronary artery with stenosis, and the myocardial segment(s) with LGE. However, because of the obvious difficulties in some cases to correctly assess which myocardial segments are supplied by a certain coronary artery we also performed a secondary, less conservative “near match” analysis. Near match was considered to exist even when LGE occurred in any adjacent segment to segments that were deemed to be supplied by a coronary artery with stenosis, with one exception. The border between septum and the free wall was angiographically clear and therefore the segments on different sides of this border were not considered near. The list of segments used when converting “exact match” to “near match” is shown in Additional file 1: Table S1.

### Statistical analysis

For statistical analysis SAS 9.3 was used. Level of significance set at *p* < 0.05. Fishers exact test was used to compare categorical values for patients. Data by segment were analysed with a generalized estimating equation (GEE) model [15] that fully accounts for any dependence between segments for the same patient. This approach approximately addresses error estimates in the context of correlated observations. In addition to the univariate model, the relation between severity of stenosis and UMI was also analysed in a multivariate model adding age, gender, diabetes and hypertension as covariates. Age was analysed as a continuous variable. Gender, diabetes and hypertension were analysed as categorical variables. Results were presented as odds ratios (OR) with 95 % confidence intervals (CIs) and p-values. When describing clinical characteristics number (%), mean and standard deviation or median and interquartile range (IQR) were used as appropriate.

## Results

### Clinical characteristics and prevalence of UMI

UMIs were found in 58 patients (24.7 %), out of whom 3 patients had two distinctly separate areas of UMI. The background characteristics of the study population stratified in those with and without UMIs are shown in Table 1. UMIs tended to be more common among men compared to women, 44/155 (28 %) vs. 14/80 (18 %) (*p* = 0.08); and in patients with compared to without diabetes mellitus, 17/49 (35 %) vs. 41/186 (22 %) (*p* = 0.09). There was no significant difference in left ventricular ejection fraction, 66 % vs. 67 % (*p* = 0,55).

**Table 1:** Background characteristics

	No UMI (*n* = 177)	UMI (*n* = 58)	*P*-value
Age at inclusion, years	64.2 ± 8.8	66.6 ± 8.2	0.07
Women (%)	66 (37 %)	14 (24 %)	0.08
Current smoker (%)	16 (9 %)	6 (10 %)	0.80
Previous smoker (%)	89 (50 %)	32 (55 %)	0.55
Family history of IHD (%)	87 (49 %)	30 (52 %)	0.76
Hypertension (%)	94 (53 %)	38 (66 %)	0.13
Diabetes mellitus (%)	32 (18 %)	17 (29 %)	0.09
Previous stroke/TIA (%)	7 (4.0 %)	6 (10 %)	0.09
PVD (%)	6 (3.4 %)	6 (10 %)	0.08
COPD (%)	13 (7.3 %)	3 (5.2 %)	0.77
BMI	27.3 ± 3.5	27.7 ± 4.0	0.49
Waist circumference, cm	99.1 ± 10.0	101.8 ± 11.7	0.55
Systolic BP, mmHg	139 ± 17	143 ± 19	0.22
Diastolic BP, mmHg	79 ± 10	79 ± 10	0.96
Ejection fraction, % median (IQR)	66 (61–71)	67 (62–72)	0.55
Symptoms of angina pectoris			
<2 months (%)	6 (3 %)	1 (2 %)	0.75
2-12 months (%)	80 (45 %)	25 (43 %)	
>12 months (%)	91 (51 %)	32 (55 %)	
Medications			
Aspirin (%)	158 (89 %)	53 (91 %)	0.81
Clopidogrel (%)	4 (2 %)	3 (5 %)	0.37
Statin/other lipid lowering agent (%)	121 (68 %)	46 (79 %)	0.13

*IHD* Ischemic Heart Disease, *TIA* Transient Ischemic Attack, *PVD* Peripheral Vascular Disease, *COPD* Chronic Obstructive Pulmonary Disease, *BMI* Body Mass Index, *BP* Blood Pressure, *IQR* InterQuartileRange

Areas of LGE without a subendocardial component, i.e. not fulfilling the criteria for UMI, were present in 25 patients (11 %).

**Fig. 2:**
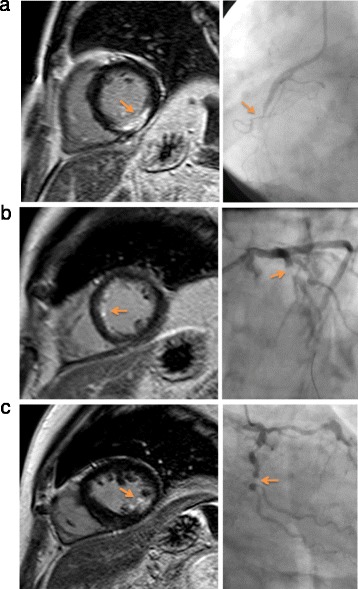
Representative images of UMIs and corresponding coronary angiographies. **a** transmural UMI in AHA segment 4 but also partly in segment 3 and 5 with corresponding RCA occlusion on coronary angiography, **b** subendocardial UMI in AHA segment 8 and 9 with corresponding high grade stenosis in proximal LAD, **c** subendocardial UMI in AHA segment 11 with corresponding high grade stenosis in proximal LCX. Three supplemental moving images of example (**c**) are also provided; Additional files 2, 3 and 4

Some representative images of UMIs and corresponding coronary angiographies are shown in Fig. 2. Additional files 2, 3 and 4 contains moving images, Additional file 2: short axis 3D CMR, Additional file 3: coronary angiography of LCA, Additional file 4: coronary angiography of RCA.

### Size and localization of the UMIs

The LGE was transmural in 21 patients, and subendocardial in 37 patients. The three patients with two distinct UMIs had in two cases two separate subendocardial UMIs and in one case one subendocardial UMI and one transmural UMI. The majority of the UMIs were small, the median size of the UMI was 2.1 g (IQR 0.7 – 4.5 g), and the maximum size was 27.8 g (Additional file 5: Figure S1).

**Fig. 3:**
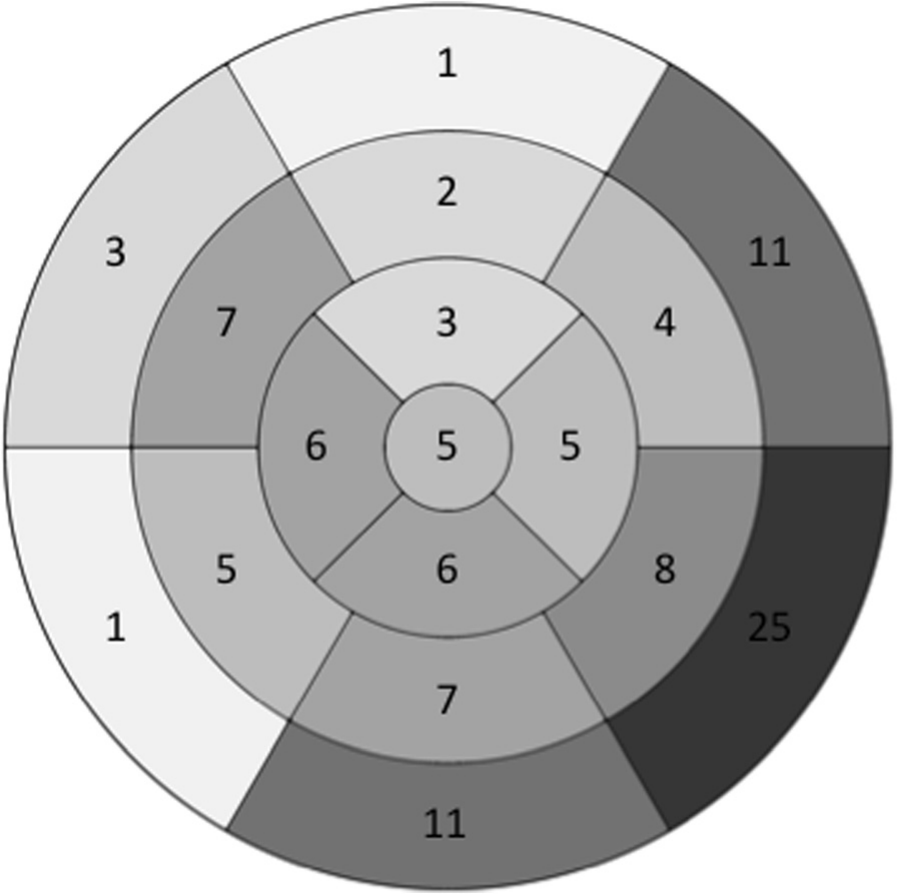
The localization of the infarctions found by CMR in the 58 patients with unrecognized myocardial infarctions (UMIs). The number in each of the 17 different myocardial segments gives how often a certain segment was affected. In total, 110 segments were affected in the 58 patients

In total 110 myocardial segments were affected in the 58 patients with UMI. The localization of the segments with UMI is shown in Fig. 3. The UMIs were predominately located in the inferior and inferior-lateral myocardial segments (AHA segments 4, 5, 10, 11, 15, 16) with 56 % of the UMIs located in those areas.

### Findings at coronary angiography

**Fig. 4:**
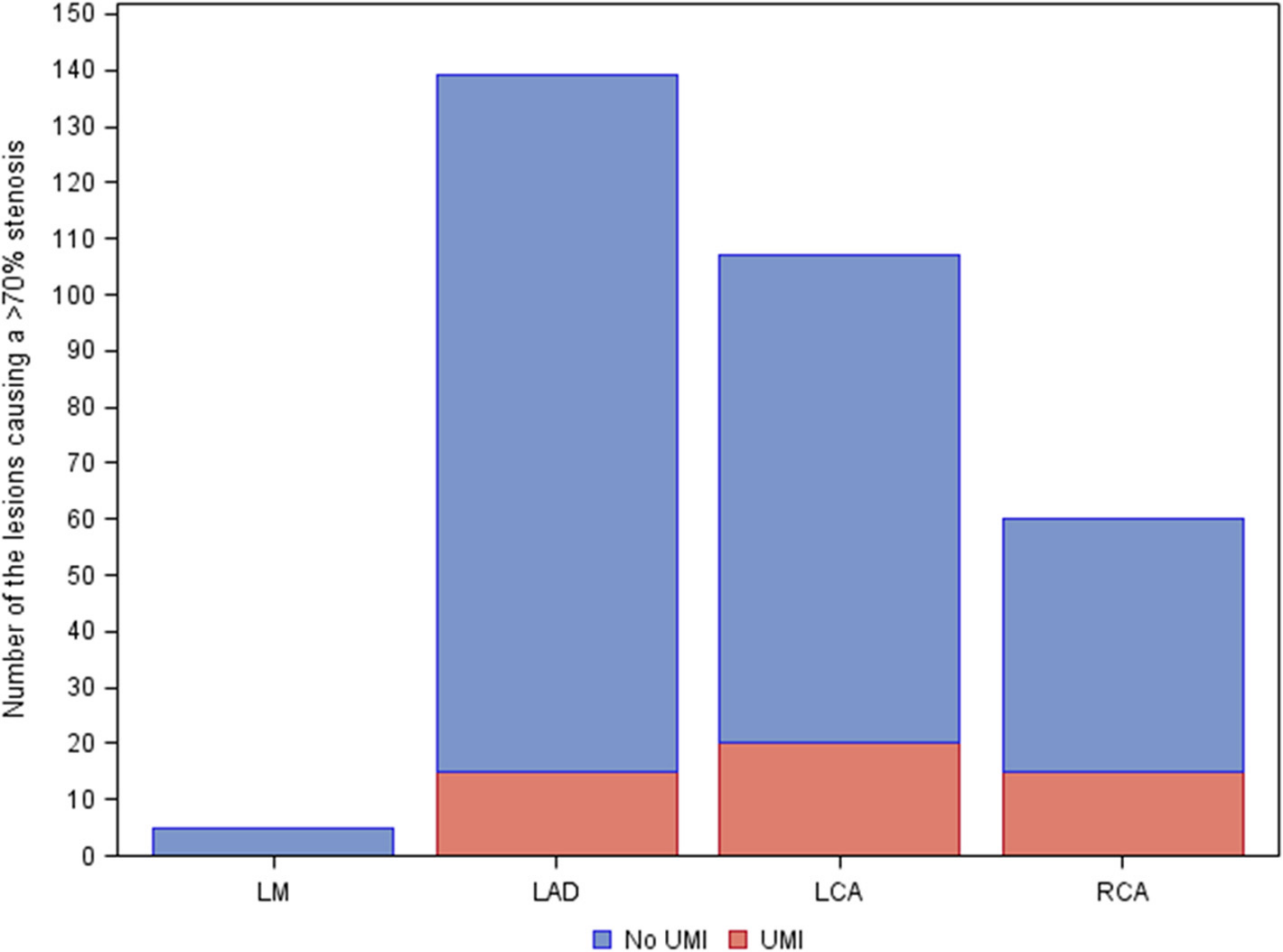
Distribution of segments supplied by a coronary artery with a stenosis ≥70 % on coronary angiography. The red part of the bars are segments with a UMI and the remaining blue bars are segments without UMI. LM = left main artery, LAD = left anterior descending artery, LCA = left circumflex artery, RCA = right coronary artery

At coronary angiography 32 patients (13.6 %) had a total occlusion of at least one coronary branch, 103 patients (43.8 %) had a maximal stenosis of ≥70-99 %, 8 patients (3.4 %) a maximal stenosis ≥50-69 %, 63 patients (30.2 %) a maximal stenosis of ≥30-49 %, and 29 patients (12.3 %) had no stenosis or a maximal stenosis grade of less than 30 %. In men, 64 % had a maximal stenosis ≥ 70 %, whereas only 45 % in women (*p* = 0.008). In patients with diabetes 80 % had a maximal stenosis ≥ 70 %, compared with 52 % in patients without diabetes (*p* < 0.0001). The distribution of the lesions causing a stenosis ≥70 % showed predominance for lesions in the LAD territory (Fig. 4), except for patients with diabetes where there was an equal predominance for LAD and LCX territories.

### Relation between coronary stenosis and UMI on patient level and on segment level

UMIs were more prevalent in the 135 patients with at least one coronary artery stenosis ≥ 70 %, as compared to the 100 patients with coronary artery stenosis <70 %, 47 (34.8 %) vs. 11 (11.0 %) (*p* < 0.0001).

When assessing the prevalence of UMI in myocardial segments downstream a coronary stenosis, taking the individual coronary anatomy in consideration and requiring “perfect match” (see method section) a significant relation between the stenosis grade in the supplying artery and the prevalence of UMI was found (Table 2). The prevalence of UMIs in segments supplied by an artery with <30 % stenosis was 1.3 % compared to 20.1 % in those segments supplied by an artery with total occlusion. It was significantly more likely to have an UMI downstream a stenosis ≥70 % as compared to <70 % (OR 5.1, CI 3.1-8.3, *p* < 0.0001). The strong association remained almost unchanged after adjustment for age, gender, diabetes and hypertension (OR 4.7, CI 2.8-7.7, *p* < 0.0001). If the segments supplied by an artery with total occlusion were excluded, it was still significantly more likely to have an UMI downstream a stenosis ≥70-99 % as compared to <70 % (adjusted OR 2.88, CI 1.6-5.1, *p* = 0.0002).

**Table 2:** Myocardial segments with and without UMI grouped by degree of coronary artery stenosis

	No UMI	UMI	Total no of segments
**Stenosis < 30 %**	1331	18	1349
	98,7 %	1.3 %	
**Stenosis ≥ 30 % -- < 50 %**	1194	14	1208
	98.8 %	1.2 %	
**Stenosis ≥ 50 % -- < 70 %**	310	3	313
	99.0 %	1.0 %	
**Stenosis ≥ 70 % -- 99 %**	927	44	971
	95.5 %	4.5 %	
**Stenosis 100 %**	123	31	154
	79.9 %	20.1 %	
**Total no of segments**	3885	110	3995

Small numbers indicate number of segments and the percentage is calculated towards the total number in the right-most column. The percentage in bold indicate the percentage of affected segments with UMI

*UMI* Unknown Myocardial Infarction, *No UMI* No Unknown Myocardial Infarction

The proportions of UMI downstream a stenosis ≥70 % tended to be unevenly distributed between RCA, LCX and LAD, 25.0 %, 18.7 % and 10.8 %, respectively (*p* = 0.08), Fig. 4. However, the size of the UMIs downstream a stenosis ≥70 % was not significantly different between the three coronary arteries (median 3.9, 3.9 and 2.9 g, respectively).

Of all myocardial segments with UMI, 68 % occurred in segments downstream a stenosis ≥70 %; no difference in proportions was found between men and women or between patients with and without diabetes.

In a sensitivity analysis, the relation between coronary stenosis and UMI requiring “near match”, but not “perfect match”, between the supplying artery with stenosis and the myocardial segment with UMI was assessed. In that analysis the proportion of myocardial segments with UMI downstream a stenosis ≥70 % rose from 68 % to 88 %. In contrast to the UMIs, areas of LGE without a subendocardial component had no clear relation to the findings at the coronary angiogram (data not shown).

## Discussion

In this multicenter study performed to prospectively evaluate the prevalence of UMI in patients with symptoms of angina pectoris and the association between UMIs and significant coronary artery disease, we could confirm a high prevalence of UMI and report two major novel findings. First, a strong association between stenotic lesions (≥70 % stenosis) in a coronary artery and the presence of an UMI in the myocardial segments supplied by that stenotic artery, taking the individual coronary anatomy in consideration. Second, a striking predominance for the UMIs to be located in the inferior and inferior-lateral myocardial segments, despite predominance for stenotic lesions in the left anterior descending artery (LAD) territory.

### Prevalence of UMI and its relation to clinical characteristics

The prevalence of UMI of 25 % in the present study is close to the 27 % found in a previous study of UMI in a similar population [8], but higher than the prevalence of 20 % [7] and 17 % [5] found in general populations, which is not surprising given the higher pre-test probability of ischemic heart disease in our study population with symptoms of angina pectoris. Consequentially, we found an association between conventional cardiovascular risk factors and occurrence of UMI (Table 1) in contrast to in the study in the general population of Barbier et al. [7].

The large difference numerically (but only reaching borderline significance) in prevalence of UMI between men and women; and patients with and without diabetes, is in line with previous studies [6, 16]. One likely explanation for these findings is the clear differences in occurrence and severity of underlying coronary atherosclerosis in the present study between men and women, and patients with and without diabetes, respectively.

### Size and localization of the UMIs

The UMIs were predominantly subendocardial infarctions and rather small, which is in accordance with previous findings [7]. However, it is important to notice that a Q-wave in the qualifying ECG was an exclusion criteria, regardless of whether the Q-wave was associated with a known previous MI or not. Nevertheless, a significant proportion of the UMIs were transmural infarctions.

In striking contrast to what is seen in recognized MIs [17], the UMIs were located predominantly in the inferior and inferolateral areas of the myocardium, in line with the findings in the study of Barbier et al. [7], but somewhat in contrast to the study by Kim et al. [8]. This distribution occurred despite that a ≥70 % stenosis was more common in the LAD territory than in the RCA and LCX territories. Hence, UMI tended to be relatively more common downstream a ≥70 % stenosis in the RCA and LCX compared to downstream a ≥70 % stenosis in the LAD (Fig. 4). The reason for this paradox is unclear, but it is likely that ischemia in the inferior and inferolateral areas of the myocardium leading to small infarctions may cause less severe, and more often atypical (e.g. abdominal pain or discomfort), symptoms and therefore not prompt the patient to seek medical attention. Hence, these infarctions may more often go undetected.

### Relation between coronary stenosis and UMI

Patients with a ≥70 % coronary stenosis had significantly more often UMI than patients without coronary stenosis in the present study, which is in line with the findings of Kim et al. [8]. Likewise, have Choi et al. [18] shown high prevalence of not only RMI, but also of UMI, downstream a chronically occluded coronary artery. However, a unique and novel feature of the present study was the attempt to evaluate in each patient individually the relation between presence of UMI and the occurrence and severity of atherosclerotic lesions in the coronary artery supplying the affected myocardial segment. Thereby, we could demonstrate a strong association between occurrence of a ≥70 % stenosis in the coronary artery and presence of UMI in the myocardial segments supplied by that coronary artery downstream of the stenosis. One could argue that this finding is driven largely by the occluded arteries since 20.1 % of segments in these territories were associated with UMI compared with only 4.5 % in those with lesions of >70-99 % and 1.0-1.3 % for less severe disease. However, a significant association remained, albeit weaker, also if the segments supplied by an artery with total occlusion were excluded. Therefore, a “dose response” effect seems to exist between the severity of stenosis and presence of UMI.

Since it is difficult to exactly match the coronary artery with the corresponding myocardial segment it supplies due to the large individual differences in coronary anatomy our finding that 68 % of the UMIs were supplied by an artery with ≥70 % stenosis is probably an underestimation. We therefore performed a secondary sensitivity analysis requiring only “near match”; in that analysis, which most likely is an overestimation, 88 % of the UMIs were supplied by a coronary artery with ≥70 % stenosis.

There is an ongoing discussion in the extent to which myocardial infarctions occur downstream an unstable plaque in a moderate stenosis or if the stenosis underlying myocardial infarctions usually are severe [19]. A recent study [20] showed fewer myocardial infarctions on follow-up in a group of patients with non-significant (FFR >0.8) coronary artery stenosis compared to a group of patients with significant coronary artery stenosis (FFR ≤ 0.8) supporting the importance of the severity of the stenosis. The results of the present study also support the importance of stenosis severity, at least for the development of UMI. The relative importance of atherosclerotic plaque rupture (myocardial infarction type 1) [21] versus supply–demand mismatch (myocardial infarction type 2) [21] as the cause of the infarction may well vary between clinically recognized MIs and UMIs. A substantial proportion of the UMIs may well be due to supply–demand mismatch, as patients with a hemodynamically significant stenosis undoubtedly are at risk of having episodes with supply–demand mismatch in the presence of a triggering factor, e.g. tachycardia. Furthermore, the small size of the UMIs may also indicate that supply–demand mismatch may not be an uncommon cause of UMI, since myocardial infarctions type 2 are usually substantially smaller than myocardial infarctions type 1 [22].

### Limitations

This is a multicenter study with sites using different CMR scanners. The examination protocols were therefore not exactly the same for all examinations. However, all CMR scans were evaluated at the core lab; only 19 CMR studies had to be excluded due to poor image quality, the remaining studies were of satisfactory quality and could be evaluated. The lesions described had to be seen in at least two planes to be accepted as representing a true finding and not an artefact.

The coronary artery stenoses were assessed visually and not using quantitative coronary angiography (QCA) grading of the lumen narrowing, nor were the stenosis evaluated by fractional flow reserve measurements, a method which has been shown to more accurately predict the hemodynamic significance of coronary stenosis [23]. Other factors that might be of importance for the risk of myocardial infarction such as the complexity of the coronary artery [24] or the coronary microcirculation [25] were not evaluated.

The assessment of which myocardial segments were affected by a coronary artery stenosis was determined subjectively taking the individual coronary anatomy into account. Unfortunately, there exist no objective or universally accepted criteria for how this should be done. However, we believe that our approach was more accurate than assuming the standardized distribution suggested by Cerqueira et al. [9] which has been shown not always to be accurate [13, 14].

## Conclusions

UMIs are common in patients with stable angina and the results indicate that the majority of the UMIs are of ischemic origin due to severe coronary atherosclerosis and in contrast to what is seen in recognized myocardial infarctions, to be predominately located in the inferior and infero-lateral myocardial segments.

## Additional files

Additional file 1:
**Table S1.** List of segments used when converting "exact match" to "near match". (DOCX 14 kb) Additional file 2:
**Moving images, short axis 3D CMR.** (MOV 3252 kb) Additional file 3:
**Coronary angiography of left coronary artery.** (MOV 18497 kb) Additional file 4:
**Coronary angiography of right coronary artery.** (MOV 17215 kb) Additional file 5:
**Figure S1.** All UMIs plotted according to size in grams (g). (PPTX 99 kb)

## Abbreviations

LGE: Late gadolinium enhancement
CMR: Cardiovascular magnetic resonance
UMI: Unrecognized myocardial infarction
MI: Myocardial infarction
ECG: Electrocardiography
PUMI: Prevalence and prognostic value of unrecognized myocardial injury in stable coronary artery disease (study)
PCI: Percutaneous coronary intervention
CABG: Coronary artery bypass graft
GFR: Glomerular filtration rate
SSFP: Steady state free precession
TR: Repetition time
TE: Echo time
CH: Chamber, as in 2CH, 3CH, 4CH
AHA: American Heart Association
PACS: Picture archiving and communications system
SCAAR: Swedish Coronary Angiography and Angioplasty Registry
LAD: Left anterior descending artery
RCA: Right coronary artery
LCX: Left circumflex artery
GEE: Generalized estimating equation
OR: Odd’s ratio
CI: Confidence interval
IQR: Interquartile range

## References

[1] Sheifer SE, Manolio TA, Gersh BJ (2001). Unrecognized myocardial infarction. Ann Intern Med.

[2] Sigurdsson E, Thorgeirsson G, Sigvaldason H, Sigfusson N (1995). Unrecognized myocardial infarction: epidemiology, clinical characteristics, and the prognostic role of angina pectoris. The Reykjavik Study. Ann Intern Med.

[3] Kannel WB, Sorlie P, McNamara PM (1979). Prognosis after initial myocardial infarction: the Framingham study. Am J Cardiol.

[4] Kim RJ, Chen EL, Lima JA, Judd RM (1996). Myocardial Gd-DTPA kinetics determine MRI contrast enhancement and reflect the extent and severity of myocardial injury after acute reperfused infarction. Circulation.

[5] Schelbert EB, Cao JJ, Sigurdsson S, Aspelund T, Kellman P, Aletras AH (2012). Prevalence and prognosis of unrecognized myocardial infarction determined by cardiac magnetic resonance in older adults. JAMA.

[6] Barbier CE, Nylander R, Themudo R, Ahlström H, Lind L, Larsson EM (2011). Prevalence of unrecognized myocardial infarction detected with magnetic resonance imaging and its relationship to cerebral ischemic lesions in both sexes. J Am Coll Cardiol.

[7] Barbier CE, Bjerner T, Johansson L, Lind L, Ahlström H (2006). Myocardial scars more frequent than expected: magnetic resonance imaging detects potential risk group. J Am Coll Cardiol.

[8] Kim HW, Klem I, Shah DJ, Wu E, Meyers SN, Parker MA (2009). Unrecognized non-Q-wave myocardial infarction: prevalence and prognostic significance in patients with suspected coronary disease. PLoS Med.

[9] Cerqueira MD, Weissman NJ, Dilsizian V, Jacobs AK, Kaul S, Laskey WK (2002). American Heart Association Writing Group on Myocardial Segmentation and Registration for Cardiac Imaging. Standardized myocardial segmentation and nomenclature for tomographic imaging of the heart. A statement for healthcare professionals from the Cardiac Imaging Committee of the Council on Clinical Cardiology of the American Heart Association. Circulation.

[10] Hunold P, Schlosser T, Vogt FM, Eggebrecht H, Schmermund A, Bruder O (2005). Myocardial late enhancement in contrast-enhanced cardiac MRI: distinction between infarction scar and non-infarction-related disease. AJR Am J Roentgenol.

[11] Austen WG, Edwards JE, Frye RL, Gensini GG, Gott VL, Griffith LS (1975). A reporting system on patients evaluated for coronary artery disease. Report of the Ad Hoc Committee for Grading of Coronary Artery Disease, Council on Cardiovascular Surgery, American Heart Association. Circulation.

[12] Velders MA, James SK, Libungan B, Sarno G, Fröbert O, Carlsson J (2014). Prognosis of elderly patients with ST-elevation myocardial infarction treated with primary percutaneous coronary intervention in 2001 to 2011: A report from the Swedish Coronary Angiography and Angioplasty Registry (SCAAR) registry. Am Heart J.

[13] Pereztol-Valdés O, Candell-Riera J, Santana-Boado C, Angel J, Aguadé-Bruix S, Castell-Conesa J (2005). Correspondence between left ventricular 17 myocardial segments and coronary arteries. Eur Heart J.

[14] Ortiz-Pérez JT, Rodríguez J, Meyers SN, Lee DC, Davidson C, Wu E (2008). Correspondence between the 17-segment model and coronary arterial anatomy using contrast-enhanced cardiac magnetic resonance imaging. JACC Cardiovasc Imaging.

[15] Zeger SL, Liang KY (1986). Longitudinal data analysis for discrete and continuous outcomes. Biometrics.

[16] Kwong RY, Sattar H, Wu H, Vorobiof G, Gandla V, Steel K (2008). Incidence and prognostic implication of unrecognized myocardial scar characterized by cardiac magnetic resonance in diabetic patients without clinical evidence of myocardial infarction. Circulation.

[17] Geske JB, Edwards WD, MacDonald RJ, Holmes DR (2010). Location of coronary culprit lesions at autopsy in 41 nondiabetic patients with acute myocardial infarction. Am J Forensic Med Pathol.

[18] Choi JH, Chang SA, Choi JO, Song YB, Hahn JY, Choi SH (2013). Frequency of myocardial infarction and its relationship to angiographic collateral flow in territories supplied by chronically occluded coronary arteries. Circulation.

[19] Niccoli G, Stefanini GG, Capodanno D, Crea F, Ambrose JA, Berg R (2013). Are culprit lesions severely stenotic?. JACC Cardiovasc Imaging.

[20] De Bruyne B, Fearon WF, Pijls NHJ, Barbato E, Tonino P, Piroth Z (2014). Fractional flow reserve-guided PCI for stable coronary artery disease. N Engl J Med.

[21] Thygesen K, Alpert JS, Jaffe AS, Simoons ML, Chaitman BR, White HD (2012). Third universal definition of myocardial infarction. Eur Heart J.

[22] Paiva L, Providência R, Barra S, Dinis P, Faustino AC, Gonçalves L (2015). Universal definition of myocardial infarction: clinical insights. Cardiology.

[23] Fischer JJ, Samady H, McPherson JA, Sarembock IJ, Powers ER, Gimple LW (2002). Comparison between visual assessment and quantitative angiography versus fractional flow reserve for native coronary narrowings of moderate severity. Am J Cardiol.

[24] Sianos G, Morel MA, Kappetein AP, Morice MC, Colombo A, Dawkins K (2005). The SYNTAX Score: an angiographic tool grading the complexity of coronary artery disease. EuroIntervention.

[25] Herrman J, Kaski JC, Lerman A (2012). Coronary microvascular dysfunction in the clinical setting: from mystery to reality. Eur Heart J.

