# Supplementary material for: Unrecognized myocardial infarctions assessed by cardiovascular magnetic resonance are associated with the severity of the stenosis in the supplying coronary artery
Source: J Cardiovasc Magn Reson. 2015 Nov 19;17:98. doi: 10.1186/s12968-015-0202-5 (PMC4653938; doi:10.1186/s12968-015-0202-5)
Supplement: Additional file 1: — Table S1. List of segments used when converting "exact match" to "near match". (DOCX 14 kb) [file 12968_2015_202_MOESM1_ESM.docx]

Supplemental table 1

| Exact match, myocardial segments according to Cerquiera | Near match, myocardial segments according to Cerquiera |
| --- | --- |
| 1 | 1,6,7 |
| 2 | 2,3,8 |
| 3 | 2,3,9 |
| 4 | 4,5,10 |
| 5 | 4,5,6,11 |
| 6 | 1,5,6,12 |
| 7 | 1,7,12,13 |
| 8 | 2,8,9,14 |
| 9 | 3,8,9,14 |
| 10 | 4,10,11,15 |
| 11 | 5,10,11,12,15,16 |
| 12 | 6,7,11,12,13,16 |
| 13 | 7,12,13,16,17 |
| 14 | 8,9,14,17 |
| 15 | 10,11,15,16,17 |
| 16 | 11,12,13,15,16,17 |
| 17 | 13,14,15,16,17 |
