# Supplementary figures and images for: Unrecognized myocardial infarctions assessed by cardiovascular magnetic resonance are associated with the severity of the stenosis in the supplying coronary artery
Source: J Cardiovasc Magn Reson. 2015 Nov 19;17:98. doi: 10.1186/s12968-015-0202-5 (PMC4653938; doi:10.1186/s12968-015-0202-5)

## Slide 1
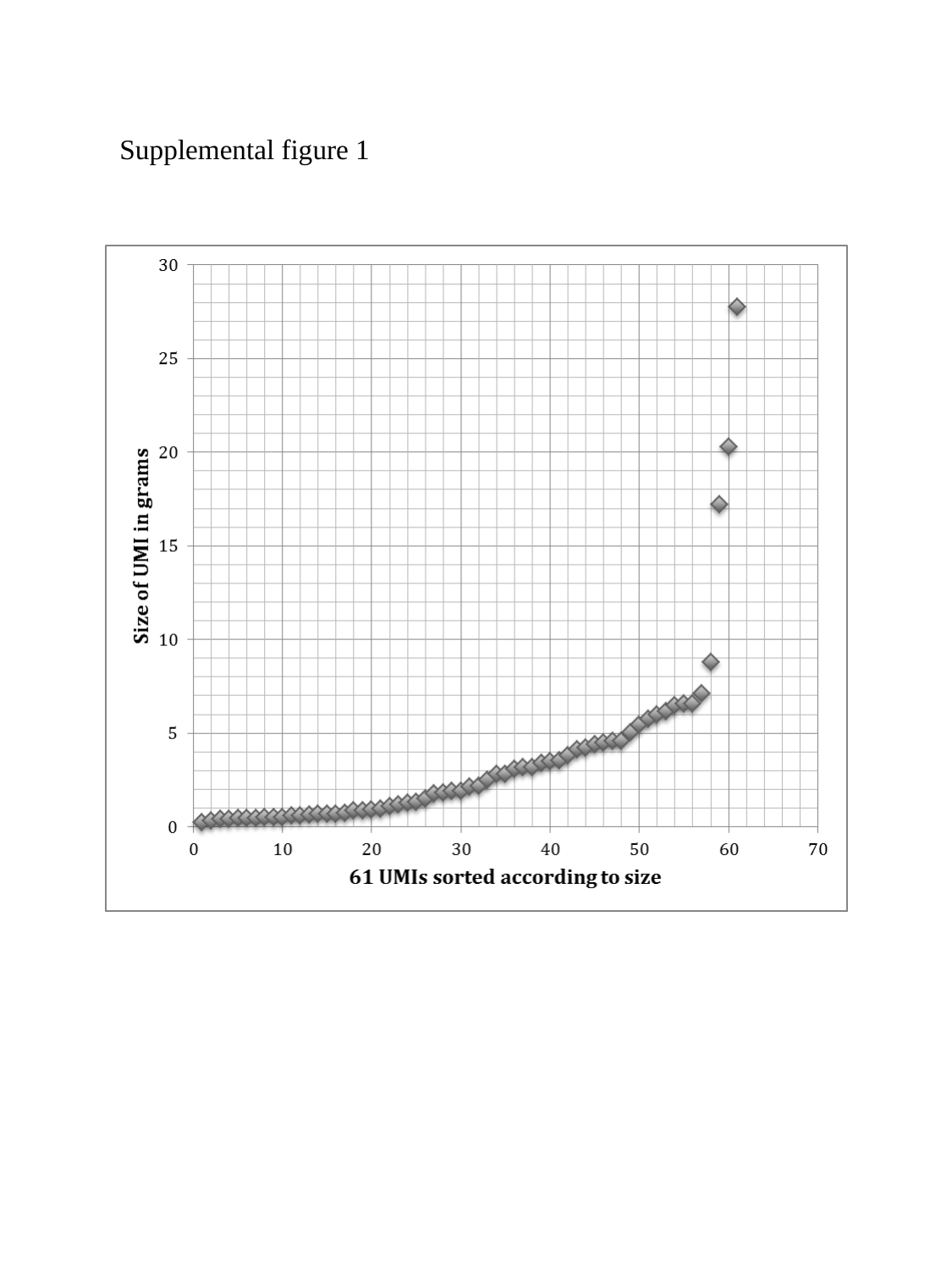

Supplemental figure 1

Supplement: Additional file 5: — Figure S1. All UMIs plotted according to size in grams (g). (PPTX 99 kb) [file 12968_2015_202_MOESM5_ESM.pptx]
